# A clinical observation of Chinese chronic myelogenous leukemia patients after discontinuation of tyrosine kinase inhibitors

**DOI:** 10.18632/oncotarget.11281

**Published:** 2016-08-13

**Authors:** Qing Li, Zhaodong Zhong, Chen Zeng, Li Meng, Chunrui Li, Yi Luo, Hongxiang Wang, Weiming Li, Jue Wang, Fanjun Cheng, Anyuan Guo, Songya Liu, Caibao Jin, Xiaojian Zhu, Yong You, Ping Zou

**Affiliations:** ^1^ Institute of Hematology, Union Hospital, Tongji Medical College, Huazhong University of Science and Technology, Wuhan 430022, P. R. China; ^2^ Department of Hematology, Tongji Hospital, Tongji Medical College, Huazhong University of Science and Technology, Wuhan 430030, P. R. China; ^3^ Department of Hematology, the Central Hospital of Wuhan, Wuhan 430016, P. R. China; ^4^ Bioinformatics and Molecular Imaging Key Laboratory, Department of Biomedical Engineering, Key Laboratory of Molecular Biophysics of the Ministry of Education, College of Life Science and Technology, Huazhong University of Science and Technology, Wuhan 430074, China

**Keywords:** discontinuation, leukemia stem cell (LSC), monitoring, microvesicle (MV), chronic myelogenous leukemia (CML)

## Abstract

Whether tyrosine kinase inhibitors (TKIs) can be safely discontinued is a key focus of chronic myelogenous leukemia (CML) at present. We report a clinical observation of TKIs cessation in Chinese CML patients and a probable connection between CML leukemia stem cells (LSCs) and relapse. In all, 22 of 1057 patients consented to participate in this observation. The average time of complete molecular response was 12.73 months after TKI withdrawal. LSCs could be flow cytometrically detected in most of the patients. However, the number of LSCs did not differ between the relapsers and non-relapsers. We evaluated the leukemogenetic ability of the LSCs by transplanting bone marrow into irradiated NOD/SCID mice. The results indicated that part of the bone marrow from the relapsers lead to leukemogensis in the mice. Besides, we found that LSCs-derived microvesicles might serve as a novel factor for the stratification of undetectable minimal residual disease and an early warning sign of relapse. In summary, post-TKI cessation relapse seems to show none association with the number of LSCs. A mouse xenograft model would provide a novel and useful method of analyzing LSCs function and predicting relapse. Microvesicles may provide important information about optimal molecular monitoring schedules in TKI discontinuation strategies.

## INTRODUCTION

Chronic myelogenous leukemia (CML) is a myeloproliferative neoplasm characterized by the constitutive expression of the oncogenic tyrosine kinase *BCR-ABL1* [1, 2]. Tyrosine kinase inhibitors (TKIs) that target *BCR-ABL1* are now the standard of care for patients with CML [1, 2]. Increasing numbers of patients who remain on TKIs for years could have undetectable minimal residual disease (UMRD), which can guarantee a long-term event-free survival and an almost nonexistent tumor burden [3, 4]. Most patients with UMRD have a strong desire to discontinue TKIs. However, a clinical cure (TKIs cessation) has not yet been proven, and life-long TKI therapy remains the consensus recommendation. In the last decade, clinical trials for the discontinuation of TKIs have consistently reported that sustained treatment-free remission (TFR) could only be observed in approximately 40% patients, with regional differences [5–8].

This raises the question of why some CML patients achieve TFR while others do not. Undoubtedly, the residual leukemia cells in patients with UMRD are responsible for the post-TKI cessation relapse. It is well known that although TKIs effectively eradicate most CML cells, they are largely ineffective in depleting quiescent leukemia stem cells (LSCs) [9, 10]. Chomel et al. performed long-term culture-initiating cell assays with CD34+ cells obtained from the bone marrow of patients with sustained undetectable molecular residual disease for 3 years or more after TKI therapy, and found *BCR-ABL1*–expressing LSCs in all cell samples [11]. To our knowledge, few researchers have highlighted the connection between LSCs and TFR after the cessation of TKI therapy. We present a clinical observation here; aim to analyze the clinical data of Chinese CML patients in whom TKI therapy had been discontinued and determine the role of LSCs in TFR after TKI discontinuation.

## RESULTS

### Outcomes of TKI discontinuation in Chinese CML patients

Only 22 of 1057 patients (2.2%) discontinued TKI therapy and were included in this clinical observation (Table 1). The median age of these 22 patients was 25 years (range, 14–68 years). The male: female ratio was 8:14. Of the 22 CML patients, 20 were in the chronic phase, and 2 were in the accelerated phase at the time of diagnosis. Calculation of Sokal scores showed that there were 9 low-risk patients, 10 intermediate-risk patients and 3 high-risk patients. All 22 patients were treated with the standard dose or a higher dose of TKIs. The average duration of TKI treatment was 72.05 months (range, 36–103 months). The reasons for cessation included patient's request due to cost (*n* = 6), patient's plan to become pregnant (*n* = 3) and long-term UMRD (*n* = 13). None of the patients received any CML-associated therapies after TKI cessation. Seven patients (32%) had received prior interferon-α (IFN-α) treatment, but none received IFN-α in combination with TKIs. The median time to major molecular response (MMR) was 9.05 months (range, 3–24 months). The median period of TKI cessation was 12.73 months (range, 1–40 months).

**Table 1:** Clinical features of the patients

No	Gender	Age (ys)	Diagnos-is	IFN be-fore TKI (m)	Time to MMR (m)	Course of TKI	Time of cessation (m)	Number Of LSC (%)	BCR-ABL1 In MV	Relapse	Treatment
1	Male	21	AP	No	3	IM 36	40	0.31	1.34	N	
2	Male	61	CP	> 12	9	IM 60 m+Ni 6 m	27	0.16	0.76	N	
3	Female	68	CP	No	3	IM 55 m	4	0.45	3.45	Y	Ni 300 mg bid
4	Female	51	CP	< 6	6	IM 84 m	2	0.66	1.96	Y	IM 400 mg qd
5	Female	20	CP	No	12	IM 95 m	22	0.76	0.85	N	
6	Female	15	CP	> 12	12	IM 84 m	20	0.07	0.55	N	
7	Female	19	AP	No	15	IM 96 m	20	0.00	0.37	N	
8	Female	29	CP	No	6	IM 66 m	18	0.55	1.32	N	
9	Female	16	CP	No	6	IM 92 m	14	0.02	1.76	Y	Refuse
10	Male	20	CP	No	6	IM 78 m	10	0.03	1.14	Y	Refuse
11	Female	34	CP	No	6	IM 72 m	13	0.30	2.98	Y	Refuse
12	Male	28	CP	No	18	IM 103 m	1	0.09	3.82	Y	IM 400 mg qd
13	Female	34	CP	No	12	IM 40 m	5	0.17	0.94	Y	Refuse
14	Female	51	CP	No	6	IM 102 m	6	0.44	0.87	Y	IM400 mg qd
15	Male	14	CP	No	15	IM 78 m	11	0.41	2.16	N	
16	Male	29	CP	> 12	24	IM 36 m+Ni 18 m	11	0.00	0.98	N	
17	Male	20	CP	No	9	IM 64 m	3	0.07	1.35	Y	IM 400 mg qd
18	Female	60	CP	No	3	IM 77 m	4	0.19	0.87	Y	IM 400 mg qd
19	Female	46	CP	> 12	6	IM 48 m+Ni 29 m	9	0.08	1.32	N	
20	Male	48	CP	No	6	IM 24 m+Ni 33 m	7	0.13	0.83	N	
21	Female	23	CP	No	7	IM 62 m	9	0.34	1.78	N	
22	Female	25	CP	< 6	9	IM 47 m	24	0.41	1.54	N	

Note: According to their Sokal scores, patients 13–15 were classified as high-risk patients, patients 2–12 were classified as intermediate-risk patients, and the rest were classified as low-risk patients.

Molecular recurrence was detected in 10 of the 22 patients during the study period. No cases of disease progression or morbidity were observed after TKI cessation. TKI treatment was restarted immediately in 6 patients, who then all recovered, with an average time to MMR of 5.33 months (range, 3–9 months). The remaining 4 patients refused to restart TKI therapy and have lost the sustained MMR for 2 months now. Interestingly, none of the 4 patients who had been treated with second-generation TKIs suffered a molecular relapse in this study (Table 1. Nos 2, 16, 19, 20).

Comparative analyses were performed to distinguish the characteristics of patients who would not suffer molecular recurrence. No significant difference was found in the median duration of imatinib therapy between the TFR group and the molecular relapse group (70.5 ± 7.7 vs. 76.7 ± 6.3, *P* = 0.54; Supplementary Figure S1A). Similarly, time to MMR (10.3 ± 1.6 vs. 7.5 ± 1.4, *P* = 0.21; Supplementary Figure S1B) and age (29.2 ± 4.3 vs. 36.4 ± 6.2, *P* = 0.34; Supplementary Figure S1C) did not differ between the two groups. Of the 22 patients, 7 received IFN-α treatment before TKIs; the rate of relapse in these patients was similar to that in patients who did not receive IFN-α treatment (3/7 vs. 8/15, *P* = 0.13). However, the 4 patients who received IFN-α treatment for 12 months or longer did not develop molecular recurrence within our observation period. In addition, molecular recurrence occurred in only 2 of 9 patients in the low-risk group, 6 of 10 patients in the intermediate-risk group and 2 of 3 patients in the high-risk group.

### Detection of LSCs

**Figure 1:**
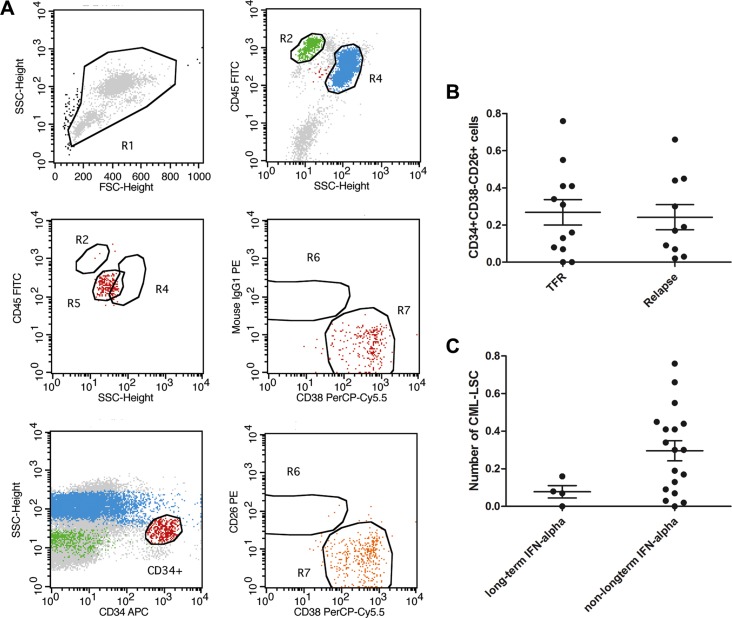
Detection of LSC by FACS (**A**) Using CD45, CD38, CD34 and CD26, we could detect a group of cells labelled as CD45+CD34+CD38−CD26+ in most of the UMRD patients by flow. (**B**) none significant difference was observed in the number of CD45+CD34+CD38−CD26+ cells (0.27% ± 0.07 vs 0.24% ± 0.07, *P* > 0.05) between the TFR group and group of molecular recurrence. (**C**) Long term of IFN-α seemed to show no impact on the number of CD45+CD34+CD38−CD26+ cells (0.08% ± 0.03 vs 0.30% ± 0.05, *P* > 0.05).

Generally, residual leukemia cells, especially LSCs, are responsible for disease relapse after TKI cessation in CML patients with UMRD. Therefore, we determined the number of CML-LSCs in the bone marrow of patients prior to the discontinuation of TKIs. Recent studies have demonstrated that the phenotype of CML-LSCs is CD34 +CD38−CD26+, with CD26+ being an important feature between normal stem cells and CML-LSCs [14, 15]. Our results showed that CD34+CD38−CD26+ cells could be detected in 20 of the 22 patients, even though these patients had achieved UMRD for years, indicating that CML-LSCs could not be eliminated by TKIs (Figure 1A). However, no significant difference was observed in the number of CD34+CD38−CD26+ cells (0.27% ± 0.07% vs. 0.24% ± 0.07%, *P* = 0.37; Figure 1B) between the TFR group and the relapse group. Interestingly, although no statistical difference was found, the number of CML-LSCs in the 4 patients who received IFN-α treatment for 12 months or longer was lower than that in the rest of the patients (0.08% ± 0.03% vs. 0.30% ± 0.05%; Figure 1C).

### Detection of microvesicles for further monitoring

**Figure 2:**
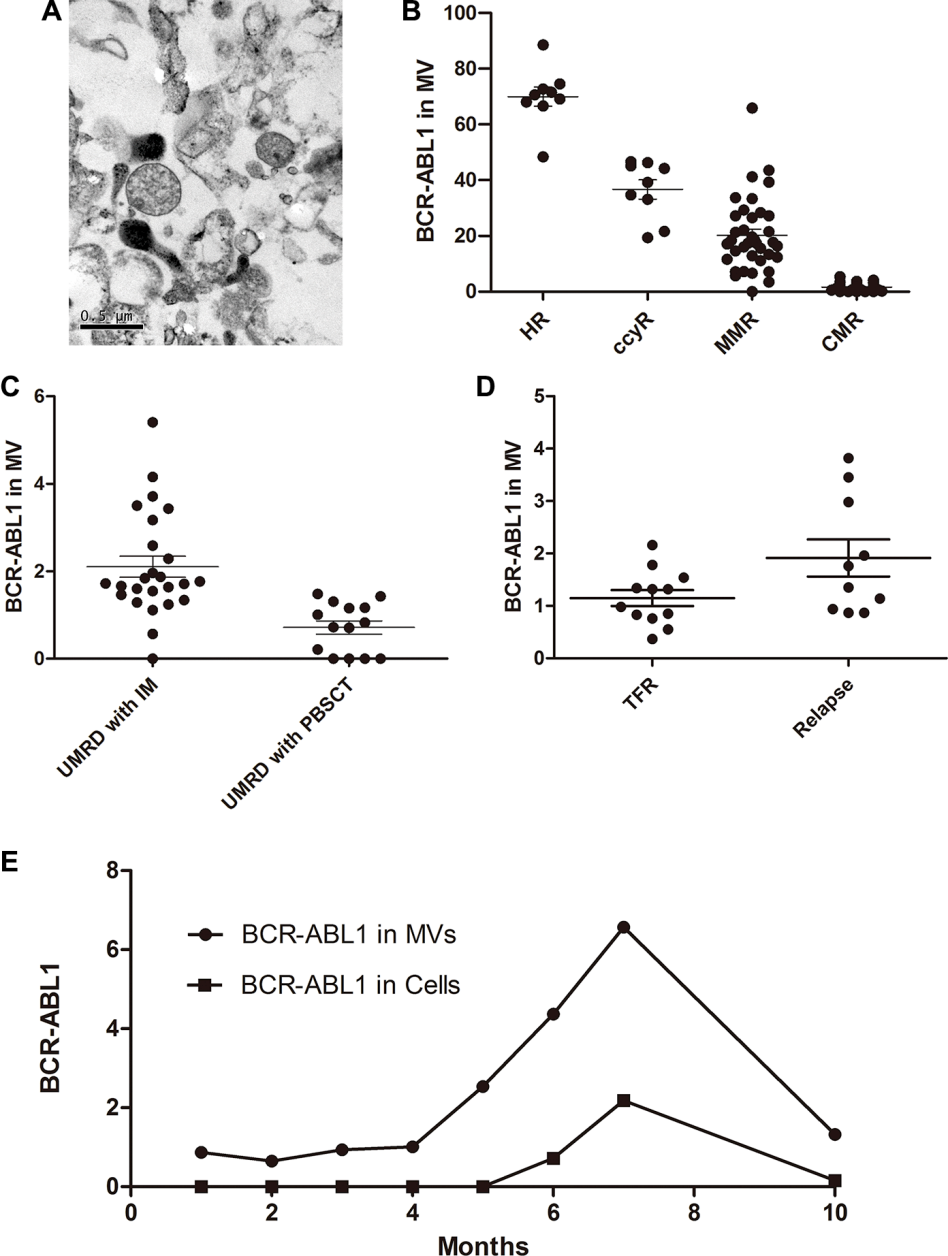
MV in the monitoring of CML patients (**A**) Scanning electron microcopy of a 1:1 mixture of 1% tungstophosphoric acid (PTA) and MV adsorped to a 300 nm mesh copper grid and stained with bromocriptine. MVs are observed as 0.3–0.8 μm vesicles in diameter (arrow). (**B**) Similar to the cellular results, *BCR-ABL1* copy number in MV was significantly different in patients with HR, CCyR, MMR and CMR. (**C**) When achieved UMRD; *BCR-ABL1* copy number in MV but not cells was significantly different in TKIs taking patients and hematopoietic stem cell transplantation recipients (2.10 ± 0.24 vs 0.72 ± 0.15, *P* < 0.05). (**D**) *BCR-ABL1* mRNA level in MV was significantly lower in TFR group than in the relapse group (1.15 ± 0.15 vs. 1.91 ± 0.35, *P* = 0.047). (**E**) Monitoring process of No.14 patient. We monitored the patients for 10 months using *BCR-ABL1* mRNA in cells and MV, respectively. In the first 4 months, *BCR-ABL1* copies in MV remained stably low meanwhile negative in cells. About one month before the positive cellular *BCR-ABL1* was detected; there was an increase of *BCR-ABL*1 copies in MV. Imatinib (400 mg qd) were given after relapse, following the treatment, copies in MV decreased.

Our previous work has demonstrated that CML cell–derived microvesicles (CML-MVs) are small membrane vesicles released by eukaryotic cells through outer cell membrane budding, and contain *BCR-ABL1* mRNA [16]. We proposed that CML-MVs could have clinical significance and prognostic relevance in the monitoring of CML patients after TKI discontinuation. To confirm that we had isolated microvesicles from the peripheral blood of the patients for RT-PCR, we examined the isolates under a transmission electron microscope. The examination revealed small vesicles with a diameter of approximately 0.5 μm (Figure 2A).

Using RT-PCR and *ABL1* as a control, we could stably detect *BCR-ABL1* mRNA in the microvesicles isolated from the peripheral blood of the patients. We discovered that *BCR-ABL1* copy numbers in the microvesicles significantly differed between CML patients with different response of TKI (Figure 2B, *P* = 0.000001), which was consistent with the results of the cellular *BCR-ABL1* mRNA tests. Sustained undetectable cellular *BCR-ABL1* on RT-PCR was defined as a CMR/UMRD, which indicated that the patient was “functionally cured”. Our data showed that copies of *BCR-ABL1* mRNA could be detected in microvesicles isolated from the peripheral blood even when the patient had achieved a CMR. Subsequently, we divided the patients who had achieved CMR into two groups: those who had been treated with TKIs and those who had undergone allogeneic hematopoietic stem cell transplantation (allo-PBSCT). Interestingly, the P210 level in the microvesicles was significantly lower in the allo-PBSCT group than in the imatinib group (2.10 ± 0.24 vs. 0.72 ± 0.15, *P* = 0.0017; Figure 2C). Among the 22 patients who discontinued TKIs, the *BCR-ABL1* level in the microvesicles was significantly lower in the TFR group than in the relapse group (1.15 ± 0.15 vs. 1.91 ± 0.35, *P* = 0.047; Figure 2D).

To investigate the role of microvesicles in patient monitoring, we detected BCR-ABL1 mRNA in the cells and in the microvesicles from the same sample of each patient once a month. In 3/6 patients who have lost TFR, durative positive *BCR-ABL1* copies in microvesicles could be observed before definitive proof of relapse. In patient 14, for example, monitoring was performed for 10 months, and involved the measurement of *BCR-ABL1* mRNA in both cells and microvesicles. In the first 3 months, both microvesicles and cells were almost negative for *BCR-ABL1* mRNA. At 6 months after cessation, BCR-ABL1 mRNA copies were in detected in the cells, and 1 month prior to this, *BCR-ABL1* mRNA was detected in the microvesicles. Imatinib (400 mg qd) was restarted immediately after the relapse (Figure 2E). Following this treatment, the mRNA copies in both microvesicles and cells decreased. In patient 12, the *BCR-ABL1* mRNA level in the microvesicles was 3.82 at the time of imatinib discontinuation. This patient suffered a rapid relapse in 1 month.

### *In vivo* evaluation of the effects of TKI cessation

**Figure 3:**
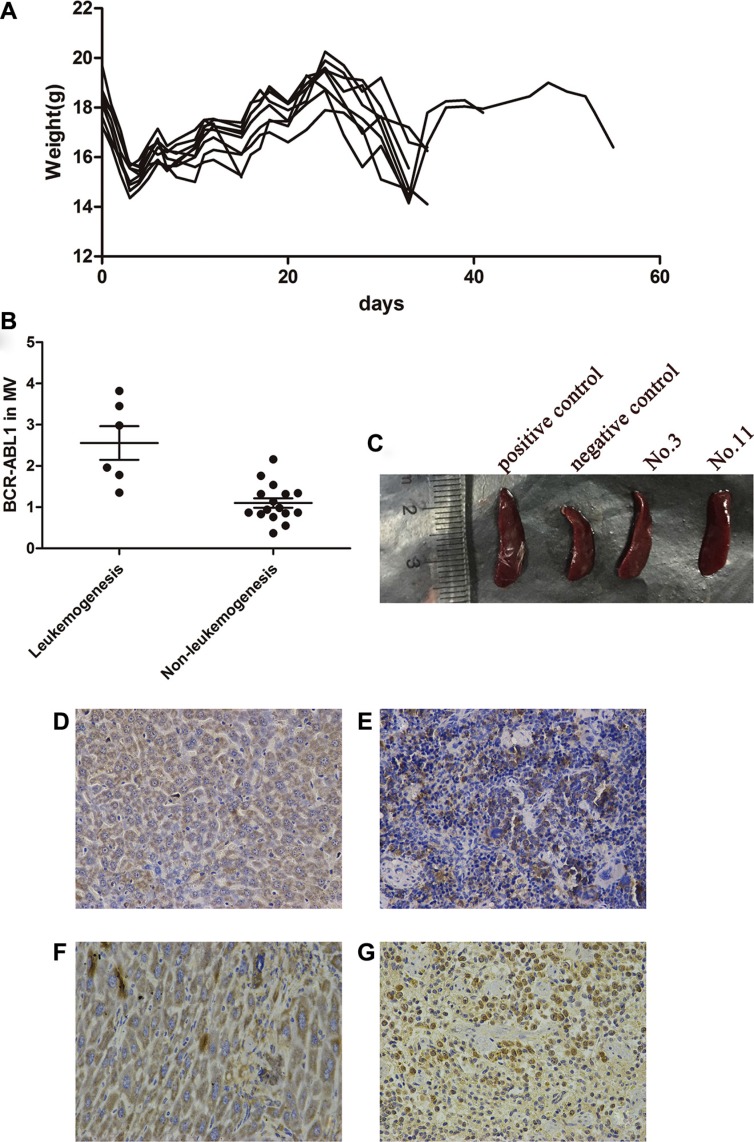
Leukemogenetic ability of the LSCs in NOD/SCID mice (**A**) Following the same tread, the 9 mice with leukemogenesis suffered a decrease in weight after irradiation at about d3. About 30 days after tail vein injection, the weight of mice decreased again and BCR-ABL1 mRNA could be detected during this period. (**B**) Higher copies of *BCR-ABL1* in MV were detected in the bone marrow with mice leukemia progression (2.56 ± 0.41 vs 1.10 ± 0.11, *P* < 0.05). (**C**) After autopsy, spleen enlargement could be observed in 6/9 mice. Immunohistochemical assay of human anti-CD45 antibodies demonstrated that the tumors contained human CD45-positive cells in liver (**D**) and spleen (**E**) of the mice. Near identical morphological features in liver (**F**) and spleen (**G**) was performed by human anti-CD38 antibodies.

Approximately 30 days after tail vein injection, 9 of 44 mice presented obviously decreased activity and weight (Figure 3A); a similar presentation was observed in the control mice transplanted with K562 cells (*n* = 5). *BCR-ABL1* mRNA was detected in the microvesicles from each bone marrow sample before transplanted into the mice. A greater number of *BCR-ABL1* mRNA copies were detected in the microvesicles from the bone marrow samples transplanted in the mice that developed leukemia progression (2.56 ± 0.41 vs. 1.10 ± 0.11, *P* = 0.015; Figure 3B). Spleen enlargement was observed on autopsy in 6 of the 9 mice transplanted with MNCs from the bone marrow of the patients (Figure 3C). Leukemia-like malignant cells could be observed in the bone marrow, spleen, liver and kidneys of the mice, and this was confirmed using immunohistochemical assays of human anti-CD45 and anti-CD38 antibodies (Figure 3D–3G). Human *BCR-ABL1* mRNA could also be detected in the bone marrow and spleen. Besides, the bone marrow samples responsible for leukemogenesis in the mice (*n* = 7) came from five patients (nos. 3, 4, 11, 12 and 17) who developed molecular recurrence and from one patient (no. 21) in the TFR group.

## DISCUSSION

The terminal goal of disease remission is to maintain stable remission after TKI cessation [17, 18]. Virtually all patients who stop TKI therapy with detectable MRD show rapidly rising *BCR-ABL1* levels; however, this may not be true of patients who stop TKIs after a period of UMRD. Many studies have reported that approximately 60% of patients who discontinue TKIs after UMRD relapse within 2–5 years [5–8], while the remaining maintain a stable UMRD. In our observation, 12/22 patients maintained a stable CMR after TKI withdrawal, and showed no evidence of CML during the study period. The relatively low rate of relapse in our study might be attributable to the short observation period and the inclusion criteria. As this was a clinical observation performed after obtaining informed consent, most of the participants would be at a lower risk than those in Stop Imatinib (STIM) trials. For example, the total course of TKI, terms of UMRD, etc., were more favorable in our study. Besides, we did not set any limit regarding the age of the patients. Thus, 7 of the 22 patients in our study were out of the usual age range in other trials (< 18 or > 60 years), and 4 of these 7 patients suffered molecular recurrence, indicating that TFR might be more difficult to maintain in these patients. Those who had a molecular relapse after discontinuation retained sensitivity to the TKIs, suggesting that discontinuation does not lead to acquired resistance. However, whether cessation raises any safety issues remains to be determined because irregular drug regimens might increase the risk of acquired mutation.

One aspect that merits further consideration is the characteristics of patients who can sustain UMRD after the discontinuation of TKIs. Retrospective surveys of clinical trials have indicated several independent factors that are associated with a lower chance of successful treatment discontinuation, for example, high risk as determined using the Sokal score and low natural killer (NK) cell count [5–8]. However, there appear to be no specific patient or disease characteristics that identify in advance those who can safely discontinue TKIs. Time to MMR, sex and total duration of TKI treatment, which have been reported to predict CMR maintenance after imatinib withdrawal in some studies [7–8], did not differ between the TFR and relapse groups in our study. This might due to that these factors do not truly reflect the probability of the progression of minimal residual leukemia: as mentioned above, LSCs might be the true cause of post-TKI cessation disease progression because they cannot be eliminated by TKIs.

We further identified the potential connection between post-TKI cessation relapse and residual CML-LSCs. Herrmann et al. demonstrated that the phenotype of CML-LSCs was CD34+CD38−CD26+, and that CD26+ was a novel feature between normal stem cells and CML-LSCs [15]. With CD34+CD38−CD26+ as the phenotype, CML-LSCs could be detected on flow cytometry in most of our patients with UMRD, which was consistent with the notion that the residual leukemia cells after TKI treatment are insensitive stem cells. However, the number of LSCs detected by flow cytometry did not differ between the TFR and relapse groups. We found that long-term IFN-α treatment might be helpful to maintain stable CMR probably because it has been reported to have the ability to kill CML-LSCs [19]. Further analysis demonstrated that long-term IFN-α and second-generation TKI treatment also had no impact on the number of residual CML-LSCs. The probable reason was that there were too few CML-LSCs to be accurately detected by flow cytometry; furthermore, we assume that the number of CML-LSCs might not be responsible for the relapse. This is why relapse after TKI cessation occurred in only a few of the patients even though residual CML-LSCs were detected in almost all patients. Mahon et al. have indicated that very similar to a reported mathematical model, relapses arise from residual leukemia cells with different proliferation kinetics (fast and slow), indicating that CML-LSCs exhibit different patterns to progression [5, 20]. In our study, 6 of the 10 patients who suffered an early relapse (within 5 months) had a similar number of CML-LSCs to that observed in patients who relapsed after 5 months, indicating that the number of CML-LSCs did not predict relapse after cessation. Thus, the key issue to achieve a clinical “cure” of CML is to identify which characteristics of CML-LSCs predict a relapse after TKI discontinuation and to develop methods to regulate this.

However, the characteristics of CML-LSCs, which are located in the bone marrow, are determined by not only their inherent properties but also the impact of the heterogeneity of the microenvironment in the bone marrow. Recent clinical retrospective reviews have demonstrated that early-relapsing patients have a different proportion and number of NK cells and T-reg cells compared to non-relapsing or late-relapsing patients [5–8]. Thus, the sorting and sequencing of LSCs could not completely reflect the cellular characteristics leading to relapse. Components of the microenvironment, especially, immune-related factors, should be taken into account when considering LSCs and relapse. We discovered that bone marrow samples from a few CML patients who had discontinued TKIs caused leukemogenesis after being transplanted in irradiated NOD/SCID mice. Since the mice did not receive TKIs, the transplanted bone marrow simulated TKI cessation *in vivo*. All of the factors of interest, such as LSCs and NK cells, could be analyzed before the bone marrow was injected into the mice in order to assess their prospective correlation with progression. More importantly, although we had many UMRD patients, only a few choose to stop TKI treatment because it is not currently possible to predict who will relapse. This is a major obstacle for the cessation research work at present. Although most of our data have been limited thus far to NOD/SCID mice, this model might to some extent serve as a convenient, operable and controllable model for the investigation of post-TKI cessation relapse as well as an early warning sign of which UMRD patients will suffer molecular recurrence if they discontinue TKIs. Consequently, this model prompts the idea that the progression of LSCs/relapses can probably be predicted and monitored by using an increasing number of related factors.

Novel methods for the further monitoring of patients with minimal residual disease are critical for treatment discontinuation trials and for identifying molecular recurrence. We proposed that CML-MVs could be suitable for patient monitoring. As a package of extracellular multi-molecular messages, microvesicles carry the proteins, lipids and nucleic acids of their parental cells, providing a potential source of disease-related biomarkers [21–23]. Our data demonstrated that the detection of *BCR-ABL1* mRNA in microvesicles by using RT-PCR could define different responses of CML patients, similar to cellular *BCR-ABL1* detection. As is known, UMRD does not guarantee the elimination of leukemia cells; A few leukemia cells persist in the bone marrow but are eliminated from the peripheral blood, resulting in a negative RT-PCR result. Interestingly, *BCR-ABL1* could be detected in the microvesicles even in patients who had achieved UMRD (defined as undetectable cellular *BCR-ABL*1 mRNA on RT-PCR). Considering that CML-LSCs persist in patients with UMRD, the circulating *BCR-ABL1*-positive microvesicles were probably derived directly or indirectly from these LSCs. As a single cell can release a large number of microvesicles, the signal of LSC could be cascade amplified by their microvesicels. Multiple studies have demonstrated that although the tumor cells themselves may be located in inaccessible sites, secreted microvesicles are able to circulate in the blood and transport information about the cancer [24], consistent with UMRD in CML. Therefore, microvesicles would be useful for CML-LSC monitoring and serve as a novel factor for the stratification of UMRD. We discovered that the P210 level in microvesicles was significantly lower in the HSCT group than in the TKI group, although all these patients had achieved UMRD as it is considered that HSCT, rather than TKIs, was responsible for the “cure” of CML. A difference in the *BCR-ABL1* level in the microvesicles might provide novel insights into this conclusion. We then investigated the role of this amplified signal from the CML-LSCs in patient monitoring after TKI cessation. The *BCR-ABL1* level in microvesicles was higher in the relapse group than in the TFR group, and durative increased *BCR-ABL1* in the microvesicles could be observed before definitive proof of relapse, indicating that *BCR-ABL1* copies in the microvesicles could be an early warning sign of relapse. The ability to release microvesicles might be one of the CML-LSC characteristics leading to relapse after TKI withdrawal.

In summary, this is the first report of Chinese CML patients who had discontinued TKI treatment. We investigated the connection between CML-LSCs and post-TKI cessation relapse. Whether or not a patient will relapse seems to depend on the function rather than the number of LSCs. A xenograft model in mice would provide a novel and useful method to analyze the function of LSCs and predict relapse. The use of microvesicles for patient monitoring probably improved the sensitivity of leukemic cell detection and provided important information about optimal molecular monitoring schedules in TKI discontinuation strategies.

## MATERIALS AND METHODS

### Inclusion criteria

Before the commencement of this study, we have observed 1057 patients who had a confirmed diagnosis of CML in the chronic or accelerated phase and were treated with TKIs at the Union Hospital, Tongji Hospital or Central Hospital between January 2000 and December 2014. The protocol of this clinical observation study was approved by the Hubei Province committee and by the institutional review board of Tongji Medical College. Furthermore, the patients provided informed consent prior to their participation in the study. The inclusion criteria were as follows: (1) a confirmed diagnosis of CML in the chronic or accelerated phase; (2) ongoing treatment with imatinib at a standard dose for at least 3 years or switching to a second-generation TKI for at least 1 year; (3) sustained CMR [4, 5] for at least 1 year before TKI cessation, (4) no history of allogeneic hematopoietic stem-cell transplantation, and (5) attendance of follow-up molecular biological tests, specifically, the quantification of *BCR-ABL1* transcripts by quantitative reverse transcription polymerase chain reaction (RT-PCR) assays of peripheral blood samples. The RT-PCR assays were performed every month in the first year, every 2 months after 1 year and every 3 months after 2 years. Both RNA extraction and RT-PCR assays of *BCR-ABL1* mRNA were performed at Hubei Province Stem Cell Research and Application Center, Union Hospital, and an authenticated RT-PCR detection center in Wuhan, China, by using the European LeukemiaNet recommendations for minimal residual disease quantification [12, 13]. In the case of a molecular relapse, patients were recommended to immediately recommence TKI treatment. Furthermore, 109 patients with different responses were included to analyze the role of microvesicles in patient monitoring. Among them, 40 and 37 patients achieved complete molecular response (CMR; median imatinib time 4 years [3–7.5 years]) and MMR (median imatinib time 3 years [1–5 yr]), respectively. 9 and 9 CML-CP patients achieved complete cytogenetic response (CCyR) and hematological response (HR), respectively. Fourteen patients (12 CP and 2 AP) received allogeneic hematopoietic stem cell transplantation (HSCT), with a median post-HSCT time of 11 months (4–26 months).

### Detection of CML-LSCs by flow cytometry

We obtained 10-ml bone marrow samples from each patient after they had provided informed consent and just prior to TKI cessation. Mononuclear cells (MNCs) were extracted from the bone marrow samples. The phenotype of CML-LSCs was analyzed using flow cytometry with fluorescein isothiocyanate-labeled antibodies to CD45, CD34, CD38 and CD26 (all from BD Pharmingen, Franklin Lakes, NJ, USA). Expression of CD26 on stem/progenitor cells (CD34+, CD45+, CD38−) was examined by multi-color flow cytometry. CD34+ cells were analysed following the recommendations and gating algorithm. Antibody reactions were controlled using isotype-matched control antibodies. Flow cytometric results obtained for CD34+/CD45+ cells and CD34+/CD26+CD45+ cells were generated and were expressed as percentage of reactive cells and ratio of median fluorescence intensities obtained with specific mAbs. The number of LSCs was determined based on the percentage of CD34-positive cells.

### Isolation of microvesicles

For microvesicle isolation, we collected 8-ml samples of the peripheral blood and bone marrow from each patient. The samples were withdrawn into three polypropylene EDTA tubes. They were centrifuged for 15 min at 1,000 × *g*. The resultant supernatant was again centrifuged at 3,000 × *g* for 40 min to remove platelets and cell sediment. The secondary supernatant was then centrifuged at 16,000 × *g* for 60 min to obtain microvesicles. To confirm that we had isolated microvesicles, we examined random samples by using transmission electron microscopy. The examination was performed as follows: the samples were stained with a mixture of 1% phosphotungstic acid and MV (ratio, 1:1), directly adsorbed on to a copper net (300 mesh), treated with bromocriptine, gently washed three times with distilled water, dried and observed under the microscope. RNA was extracted from the microvesicles by using adsorption (poly[U]-Sepharose-4B, Pharmacia, Sweden) and real-time PCR. The copy number in the microvesicles was determined using the ratio of *BCR-ABL1*/*ABL1*.

### NOD/SCID mouse model

We transplanted the bone marrow obtained from the patients into 3–4-week-old NOD/SCID (nonobese diabetic/severe combined immunodeficiency) mice. The mice were purchased from Huafukang Co. Ltd. (Beijing, China). All animal experiments were deemed ethically acceptable by the Hubei Province Animal Experiment Regulations. Before transplantation, all mice were subjected to total body irradiation with 2 Gy X-rays (300 kV, 10 mA; Philips MG-324, Hamburg, Germany). MNCs were extracted from 10 ml bone marrow of each patient and injected into two individual mice via the tail vein to avoid injection error. An equal amount of K562 cells (5 × 10^8^/mL, 0.2 mL) was injected in 5 mice as a control. At periodic intervals after transplantation (days +15 and +30), blood samples were aspirated from the mice for *BCR-ABL1* detection. At the end of the experiments (day +60), the mice were killed, and RNA was extracted from their spleens, and bone marrow cells were flushed from their femurs and tibiae. The liver and spleen cells were analyzed morphologically and by immunohistochemistry for the presence of human CD45 and CD38 antibodies in order to recognize human-specific hematopoietic lineage markers.

### Statistical analysis

Groups were compared using non-parametric analysis of variance and unpaired *t*-tests. Correlation analysis was performed using product–moment correlation coefficients. SPSS 10.0 was used for the statistical analysis. *P* < 0.05 was considered significant.

## SUPPLEMENTARY MATERIALS FIGURES


