# Supplementary material for: A clinical observation of Chinese chronic myelogenous leukemia patients after discontinuation of tyrosine kinase inhibitors
Source: Oncotarget. 2016 Aug 13;7(36):58234–43. doi: 10.18632/oncotarget.11281 (PMC5295427; doi:10.18632/oncotarget.11281)
Supplement: Supplementary file 1 [file oncotarget-07-58234-s001.pdf]

A clinical observation of Chinese chronic myelogenous leukemia patients after discontinuation of tyrosine kinase inhibitors

Supplementary Materials

RESULTS

Comparative analyses were performed to distinguish the characteristics of patients who would not suffer molecular recurrence. No significant difference was found in the median duration of imatinib therapy between the TFR group and the molecular relapse group (70.5 ± 7.7 vs. 76.7 ± 6.3, $P = 0.54$; Supplementary Figure S1A). Similarly, time to MMR (10.3 ± 1.6 vs. 7.5 ± 1.4, $P = 0.21$; Supplementary Figure S1B) and age (29.2 ± 4.3 vs. 36.4 ± 6.2, $P = 0.34$; Supplementary Figure S1C) did not differ between the two groups.

MATERIALS AND METHODS

RNA extraction and RT-PCR for BCR-ABL1 mRNA

Total RNA was extracted with E.Z.N.A.TM Blood RNA Kit (Omega Biotek Inc, Norcross, GA, USA). For reverse transcription (RT), 2 μg of total RNA was primed with a random hexamer mixture as primer using Moloney murine leukaemia virus Reverse Transcriptase (Promega Corporation, Madison, WI, USA) according to the protocol. Real-time quantitative PCR was performed by mixing 10 μl SYBR Green PCR Master Mix (Applied Biosystems Inc, Foster City, CA, USA), 2 μl primers (Invitrogen Corporation, Carlsbad, CA, USA), 6.5 μl RNase-free H2O, and 1.5 μl of cDNA as a template to a final reaction volume of 20 μl. Fluorescence intensity was measured using the Stratagene Mx3000P TM QPCR System.

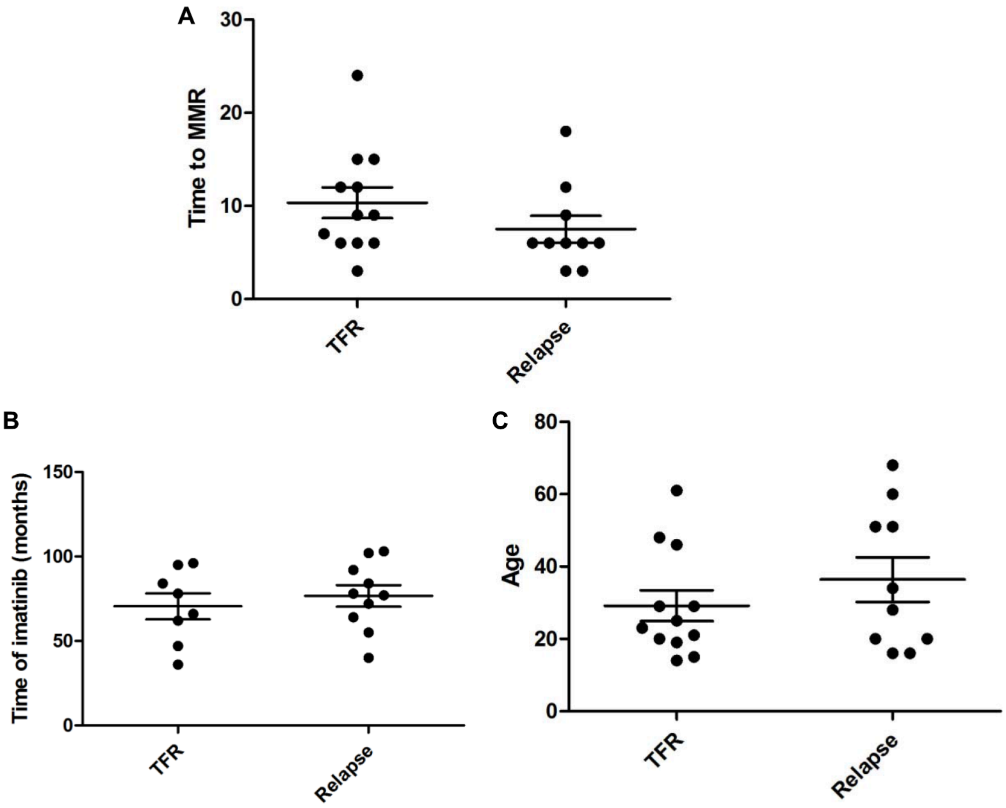

Supplementary Figure S1: Between the TFR group and the group of molecular recurrence, none significant difference was found in, time to MMR (10.3 ± 1.6 vs 7.5 ± 1.4, *P* = 0.21; (A), median course of imatinib (70.5 ± 7.7 vs 76.7 ± 6.3, *P* = 0.54, (B) and age (29.2 ± 4.3 vs 36.4 ± 6.2, *P* = 0.34; (C).
